# The glenohumeral joint - a mismatching system? A morphological analysis of the cartilaginous and osseous curvature of the humeral head and the glenoid cavity

**DOI:** 10.1186/1749-799X-9-34

**Published:** 2014-05-13

**Authors:** Valentin Zumstein, Marko Kraljević, Sebastian Hoechel, Annemarie Conzen, Andrej Maria Nowakowski, Magdalena Müller-Gerbl

**Affiliations:** 1 Institute of Anatomy, University of Basel, Pestalozzistrasse 20, Basel 4056, Switzerland; 2 Orthopädisches Zentrum München-Ost, Kreillerstrasse 156, Munich 81825, Germany; 3 Orthopedic Department, University of Basel, Spitalstrasse 21, Basel 4031, Switzerland

**Keywords:** Glenohumeral joint, Cartilage, Radius, Curvature, Geometry, Shoulder arthroplasty, Osteochondral allograft

## Abstract

**Background:**

Radial mismatch, glenohumeral conformity ratios and differences between cartilaginous and osseous radii highly depend on the measured plane. The comparison of cartilaginous radii between humeral head and glenoid in different planes provides new information to understand the degree of conformity during abduction of the upper limb.

**Methods:**

To investigate the radii, CT-images in soft-tissue kernel of 9 specimen were analysed using an image visualization software. Statistical analysis of the obtained data was performed using the *t*-test.

**Results:**

Measurements of the radii in the glenoid revealed a significantly larger radius for bone than cartilage, whereas for the humeral head the opposite was the case. Highest ratios for cartilage in the transverse plane were found in the inferior and central areas of the joint surface, whereas the smallest ratios were found in the superior area. The radial mismatch varied between 0.1 mm and 13.6 mm, depending on the measured plane.

**Conclusions:**

The results suggest that in abduction, the cartilaginous guidance of the humeral head decreases. This might permit the humeral head an anterior-posterior shifting as well as superior-inferior translation. Surgical reconstruction of the normal glenohumeral relationships necessitates precise information about the glenohumeral morphology to ensure proper sizing and correct placement of prosthetic components and osteochondral allografts.

## Background

The shoulder joint is the most mobile joint in the body and at the same time a very unstable articulation due in part to the unequal proportions of the surface areas of the glenoid compared to the humeral head. Small articulating surface areas correlate with greater probability of dislocation [1]. Shoulder instability and rotator cuff injuries represent the most common reasons of shoulder pain and dysfunction and often correlate with secondary glenohumeral osteoarthritis. The value and importance of understanding the true shape and the conformity of the glenohumeral joint are based on the evaluation of the cartilage, joint kinematics, chondral grafting, tissue engineering, and prosthetic joint replacement. To achieve reliable data about radii of curvature in the shoulder joint, it is essential not only to measure the radii in osseous structure, but to take into account the cartilaginous curvature as well. This information enables conclusions to be drawn regarding the actual biomechanical situation of the shoulder joint.

Previous studies put the focus especially on documentation of osseous anatomical characteristics like shape, inclination and version [2-4] to provide information regarding implant fixation and orientation [3,5,6]. To follow the new trend towards a more biological treatment of cartilage damage especially in younger patients, further information about the glenohumeral geometry is necessary. Since osteochondral resurfacing replaces only parts of the articulating surface, it is important to fit the allograft anatomically in the native surface to be reconstructed [7]. It is of utmost importance to know the radii of curvature in cartilaginous and bony structure in different planes in order to achieve optimal matching of the allograft and therefore best possible clinical results. Information about curvature in the glenohumeral joint is also useful when choosing the optimal prosthetic implant for shoulder replacement procedures. Recent studies investigated the influence of glenohumeral prosthetic mismatch on glenoid radiolucent lines and reported a significant relationship between mismatch and the glenoid radiolucency score [8]. Radial mismatches of 5.5 mm or more were significantly associated with lower (better) radiolucency scores.

Previous studies investigated the normal anatomical characteristics of the osseous structure in the humeral head [9-13] or the glenoid [14]. To provide reliable information about in vivo conditions it is essential to analyse not only the osseous structure, but also the cartilaginous radii of the glenohumeral joint, particularly because it is known that there exist great differences between osseous and cartilaginous radii. Therefore we investigated radii in osseous and cartilaginous structure at different planes and compared them with each other. Our hypothesis was (1) that radial mismatch, ratios and differences between cartilaginous and osseous radii highly depend on the measured plane and (2) that the comparison of cartilaginous radii between humeral head and glenoid in different planes provides new information to understand the degree of conformity during abduction of the upper limb.

## Material and methods

This study included CT-data sets of 9 fresh cadaveric shoulders from the right side (age 20–63 years, mean age 41 years, two females and seven males). The interval between death and investigation was kept to 48 hours at most. No obvious signs of degeneration or signs of joint instability (Hill-Sachs- or Bankart lesion) were observed. All experiments are in compliance with the current laws of Switzerland and with the Helsinki Declaration.

**Figure 1:**
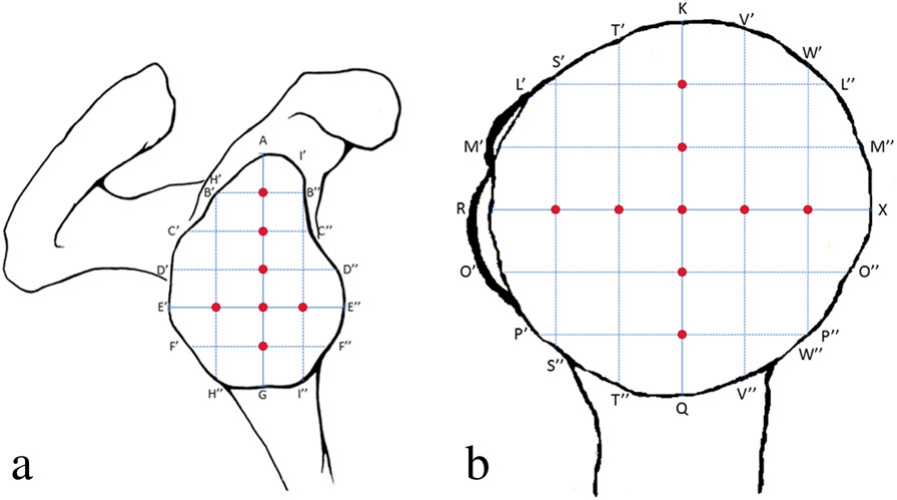
(a) Five points determined by equidistance were marked along the length axis (AG) and 3 along the transverse axis (E’E”) to determine the planes to measure the horizontal and vertical radii (B’B” - F’F” and H’H” - I’I”), (b) for the humeral head 5 points determined by equidistance were calculated for both the length (KQ) and transverse axis (RX) to determine the individual planes where radii were measured (L’L” - P’P” and S’S” - W’W”).

The specimens were scanned in an anatomical axial direction in a CT scanner (Siemens Somatom Plus 4; Slice thickness: 2.0 mm; Peak kV: 120 kV; X-ray tube current: 130 mA; Convolution kernel: 59). The obtained raw-data was reconstructed in soft-tissue kernel for on-display measurement using the image visualization software VGStudio Max 2.1.1. (Volume Graphics GmbH, Heidelberg, Germany). Surface determination and orientation of the specimens were performed using the method described by Nowakowski et al. [15]. The maximum superior-inferior and anterior-posterior distances were determined for each glenoid cavity. Then, 5 points determined by equidistance were marked along the length axis and 3 along the transverse axis to determine the planes to measure the horizontal and vertical radii. For the humeral head the greatest superior-inferior and anterior-posterior distances were obtained as well. Five points determined by equidistance were calculated for both the length and transverse axis. Based on the work of Iannotti et al. [10], we measured a best fit radius in 8 planes within the glenoid cavity (five horizontal and three vertical radii) (Figure 1a) and in 10 planes in the humeral head (five horizontal and five vertical radii) (Figure 1b). The method of best fit radius determination is a semiautomatic procedure generated by VGStudio Max 2.1.1. in accordance with the surface determination by Hounsfield units. Regarding the Gaussian distribution of Hounsfield units for bone and cartilage, the isosurface of the articular cartilage and the subchondral bone plate directly beneath the cartilage were determined as volume-surfaces. On these, a minimum of three selected points enabled the software to automatically fit a circle onto the surface from which the radius was calculated.

The measured radii in cartilaginous and bony structure were compared to each other and the obtained differences were analysed for statistical significance using the *t*-test. The level of significance was determined by *p* < 0.001.

## Results

### Plane based results of the measurement of the radii

In the glenoid, the mean values for the radii were calculated at 8 different planes within the surface area (Table 1). The greatest radii were found in the transverse plane, for bone in the central part (E’E” = 51.5 mm) and for cartilage in the superior area (B’B” = 32.5 mm). I’I” = 31.4 mm and F’F” = 22.5 mm were found to be the smallest radii for bone and cartilage and situated in the anterior and inferior areas.

**Table 1:** Mean values, ranges and differences between osseous and cartilaginous radii in the glenoid cavity

	**Bone**		**Cartilage**		
**Radius**	Mean*	Range*	Mean*	Range*	Difference*
*Transverse plane*					
B’B”	43.3	37.7	32.5	32.3	10.8†
C’C”	37.8	27.4	25.1	8.1	12.7†
D’D”	45.7	31.5	28.1	17.0	17.6†
E’E”	51.5	51.2	25.6	8.7	25.9†
F’F”	39.7	33.2	22.5	12.5	17.2†
*Coronal plane*					
H’H”	39.5	30.17	29.2	11.1	10.3†
AG	33.7	13.3	28.2	6.8	5.5†
I’I”	31.4	13.0	26.2	8.4	5.2†

^†^Statistically significant.

*in mm.

In the humeral head, the radius was measured at 10 different planes and revealed greatest values at KQ = 24.7 mm for bone and KQ = 25.4 mm for cartilage (Table 2), confirming that the greatest radii were found to be in the coronal plane most notably in central areas of the articular surface. Inferior parts of the humeral head demonstrated the smallest radii for both bone (P’P” = 17.9 mm) and cartilage (P’P” = 18.5 mm).

**Table 2:** Mean values, ranges and differences between osseous and cartilaginous radii in the humeral head

	**Bone**		**Cartilage**		
**Radius**	Mean*	Range*	Mean*	Range*	Difference*
*Transverse plane*					
L’L”	18.4	5.6	18.9	5.6	−0.5†
M’M”	22.6	4.7	23.2	4.8	−0.6†
RX	23.5	4.9	24.0	5.4	−0.5†
O’O”	22.2	4.9	22.6	5.1	−0.4†
P’P”	17.9	4.9	18.5	4.1	−0.6†
*Coronal plane*					
S’S”	18.4	3.6	19.0	4.5	−0.6†
T’T”	23.4	5.0	24.1	4.9	−0.7†
KQ	24.7	4.7	25.4	4.3	−0.7†
V’V”	23.1	4.0	23.8	3.6	−0.7†
W’W”	18.7	3.2	19.6	4.2	−0.9†

^†^Statistically significant.

*in mm.

### Differences between bone and cartilage

**Figure 2:**
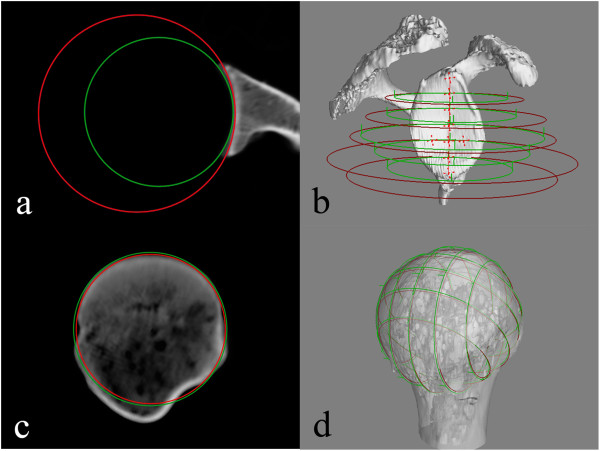
Radius measurement in the transverse plane of the (a) glenoid cavity and (c) humeral head, (b) 3D view of cartilaginous (green circle) and osseous (red circle) radii in the transverse plane of the glenoid cavity, (d) radius of curvature in the coronal and transverse plane of the humeral head.

**Figure 3:**
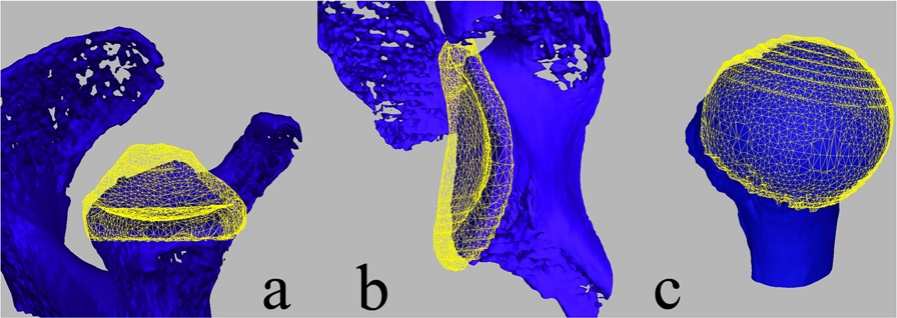
Visualisation of the cartilaginous (yellow mesh) and osseous structure (blue) in (a) infero-superior view of the glenoid, (b) antero-posterior view of the glenoid and (c) frontal view of the humeral head.

Measurements of the radii in the shoulder joint revealed differences between bone and cartilage (Figure 2 and 3). In all glenoid cavities, the osseous radius was found to be greater than the cartilaginous radius. Greatest differences were found in the transverse plane in the central parts of the glenoid (D’D”; E’E”; and F’F”) (Table 1).

In the humeral head, the radii of cartilage showed greater values than the radii of bone (Table 2).

### Mismatch and ratio between radii of the glenoid and the humeral head

The mean ratio between the radii of the glenoid cavity and the humeral head was 0.6 ± 0.1 for bone, whereas the mean ratio in cartilaginous structure was 0.9 ± 0.1. The highest ratios for cartilage in the transverse plane were found in the inferior and central areas (F’F”/O’O”; E’E”/RX), smallest ratios were found in the superior area (B’B”/L’L”) (Table 3). The radial mismatch varied between 0.1 mm and 13.6 mm, depending on the measured plane (Table 3).

**Table 3:** Mismatch and ratios of selected cartilaginous radii of the glenoid and the humeral head

**Radius glenoid/Radius humerus**	**Mismatch (mm)**	**Ratio**
*Transverse plane*		
B’B“/L’L“	13.61	0.58
D’D“/M’M“	4.90	0.83
E’E”/RX	1.57	0.94
F’F“/O’O“	0.11	1.00
*Coronal plane*		
H’H“/V’V“	5.40	0.82
AG/KQ	2.81	0.90
I’I”/T’T”	2.18	0.92

The ratio between radii RX and KQ, indicating the sphericity of the humeral head, revealed 0.95 for both bone and cartilage.

### Statistical analysis

The differences between the measured radii in cartilaginous and bony structure were examined for statistical significance using the *t*-test. The level of significance was determined by *p* < 0.001. All differences were statistically significant (Tables 1 and 2).

## Discussion

Reconstruction of the normal glenohumeral relationships necessitates precise information about the glenohumeral morphology to ensure proper sizing and correct placement of prosthetic components and osteochondral allografts [7,10].

Factors limiting application of the data obtained in this study are the relatively small number of samples and the absence of a patients group, which would allow conclusions to be drawn on biomechanical changes in diseased shoulders.

The results of our study showed significant differences regarding the plane-based results of the measurement of the radii. Concerning the horizontal plane, the surface of the glenoid cavity is much more curved in central and inferior areas, whereas superior regions showed larger cartilaginous radii and therefore indicate a less curved surface. To our knowledge, this is the first study which provides valuable measuring data of the cartilaginous and bony radii in various planes. Using embalmed cadavers, McPherson et al. [12] evaluated the osseous curvature of the glenoid using conventional 2-D radiographs. Their results described an average radius of 32.2 mm ± 7.6 mm in the anteroposterior view and 40.6 mm ± 14 mm in the axillary-lateral view. These findings indicate a more curved glenoid in the coronal plane compared to the transverse plane. In our study we provide a much more differentiated picture: Considering the measured radii in osseous structure, the findings by McPherson et al. [12] can be supported. Our measured data in cartilaginous structure, which are more important to understand the actual glenohumeral biomechanical situation than the osseous radii, show exactly the opposite pattern. The only existing study [16] providing data of cartilaginous and osseous radii is limited by the fact that only few measurements were taken which, do not resemble the actual 3-D architecture that our results show.

The humeral head showed greatest radii in the central parts. Considering cartilaginous structure, the mean radius RX in the transverse plane was 1.4 mm less than KQ in the coronal plane. The ratio between RX and KQ was 0.95, which indicates an elliptical shape of the humeral head with its greatest proportion in the superior-inferior direction. These findings are supported by the conclusions reached by Iannotti et al. [10] and several other authors [17-19].

Furthermore, we found statistically significant differences between cartilaginous and osseous radii, both in the glenoid and the humeral head. The osseous radius was found to be greater than the cartilaginous radius in all glenoid cavities. However, in the humeral head the radii of cartilage showed to be larger than osseous radii. The most noticeable differences between the radii in osseous and cartilaginous structure in the glenoid were found in the transverse plane, notably in central and inferior areas. According to the reportings of Soslowsky et al. [16], these findings can be explained by the fact that, due to the cartilaginous part of the articular surface, a certain degree of congruence between both joint partners can be achieved, especially in central and inferior parts of the glenoid cavity.

The mean ratio between the radii of the glenoid cavity and the humeral head was at a significantly higher level for the cartilaginous radii (0.9 ± 0.1) compared to those of bony structure (0.6 ± 0.1). The mismatch between the radii of the glenoid and the humeral head ranged from 0.1 mm to 13.6 mm, indicating the existence of different degrees of conformity during abduction in the shoulder joint. A study of glenohumeral mismatch [8] ranging from 0–10 mm showed that the mismatch had a significant influence on the scores for the glenoid radiolucent lines, which were best when the radial mismatch was between 6 and 10 mm. Considering this information, the mismatch measurement data obtained in several planes could play an important role in improving results of shoulder arthroplasty. Comparing cartilaginous structure of both joint partners, high ratios could be detected in coronal planes (ratios between 0.82 and 0.92). This moderate degree of congruency supports the findings reported by Graichen et al. [20], who investigated the glenohumeral translation during elevation of the shoulder and thereby reported an initial minimal translation superiorly followed by movement of the center of the head towards more inferior regions. The analysed cartilage of the glenoid herby encloses the humeral head just enough to permit the needed superior and inferior translation in abduction and adduction, but provides the needed guidance to centralize the center of rotation. Regarding the transverse plane, the obtained ratios subsequently decreased from 1.00 at inferior parts of the articular surface to 0.58 at the most superior measured radii. This new information suggests the presence of a maximum of conformity in the transverse plane in inferior and central parts of the glenoid. The articulating surface of the humeral head is highly congruent to the cartilaginous surface of the glenoid, which helps to provide an anterior or posterior shifting of the humeral head. During abduction, the superior areas of the humeral head face more superior regions of the glenoid, where the measured ratios subsequently decrease. Recent studies investigating the subchondral mineralization patterns as a marker of the loading history reported recurring patterns with anterior and posterior mineralization maxima, which might be explained by a loss of this cartilaginous guidance during abduction [21]. Favre et al. [22] detected that maximal muscle forces required for arm elevation in the scapular plane occur between 20° - 70° of abduction. Therefore, the decreasing cartilaginous guidance of the humeral head could be compensated by increased muscle force which centers the humeral head in the glenoid cavity while abduction.

## Conclusions

The results suggest that in abduction, the cartilaginous guidance of the humeral head decreases. This might permit the humeral head an anterior-posterior shifting as well as superior-inferior translation. Surgical reconstruction of the normal glenohumeral relationships necessitates precise information about the glenohumeral morphology to ensure proper sizing and correct placement of prosthetic components and osteochondral allografts.

## Competing interests

The authors declare that they have no competing interests.

## Authors’ contributions

VZ and MK carried out the the CT-scans, the image postprocessing the interpretation of the results and the writing of the manuscript. AC supported VZ and MK during the postprocessing technique. SH participated in the design of the study, performed the statistical analysis and designed the final artwork. MMG and AMN conceived of the study, and participated in its design and coordination and helped to draft the manuscript. All authors read and approved the final manuscript.
